# (1*S*,3*R*,8*R*,9*R*,10*S*)-2,2-Dibromo-3,7,7,10-tetra­methyl-9β,10β-ep­oxy-3,7,7,10-tetra­methyl­tricyclo­[6.4.0.0^1,3^]dodeca­ne

**DOI:** 10.1107/S1600536813006077

**Published:** 2013-03-09

**Authors:** Abdelouahd Oukhrib, Ahmed Benharref, Mohamed Saadi, Moha Berraho, Lahcen El Ammari

**Affiliations:** a Laboratoire de Chimie Biomoléculaire, Substances Naturelles et Réactivité "Unité Associée au CNRST (URAC16)", Faculté des Sciences Semlalia, BP 2390, Bd My Abdellah, 40000 Marrakech, Morocco; b Laboratoire de Chimie du Solide Appliquée, Faculté des Sciences, Avenue Ibn Battouta, BP 1014 Rabat, Morocco

## Abstract

The title compound, C_16_H_24_Br_2_O, was synthesized from β-himachalene (3,5,5,9-tetra­methyl-2,4a,5,6,7,8-hexa­hydro-1*H*-benzocyclo­heptene), which was isolated from the essential oil of the Atlas cedar (*Cedrus atlantica*). The mol­ecule contains fused six- and seven-membered rings, each linked to a three-membered ring. The six-membered ring has a half-chair conformation, while the seven-membered ring displays a chair conformation. The dihedral angle between the mean planes through the six- and seven-membered rings is 39.55 (12)°. The two three-membered rings, linked to the six- and seven-membered rings, are nearly perpendicular to the six-membered ring, making dihedral angles of 78.6 (2) and 80.5 (2)°, respectively. The absolute structure was established unambiguously from anomalous dispersion effects. In the crystal, each mol­ecule is linked to its symmetry-equivalent partner by C—H⋯O hydrogen bonds, forming zigzag chains parallel to [100].

## Related literature

For the isolation of β-himachalene, see: Joseph & Dev (1968 ▶); Plattier & Teisseire (1974 ▶). For the reactivity of this sesquiterpene, see: Lassaba *et al.* (1998 ▶); Chekroun *et al.* (2000 ▶); El Jamili *et al.* (2002 ▶); Sbai *et al.* (2002 ▶); Dakir *et al.* (2004 ▶). For its biological activity, see: Daoubi *et al.* (2004 ▶). For ring puckering calculations, see: Cremer & Pople (1975 ▶). For a similar structure, see: Benharref *et al.* (2010 ▶).
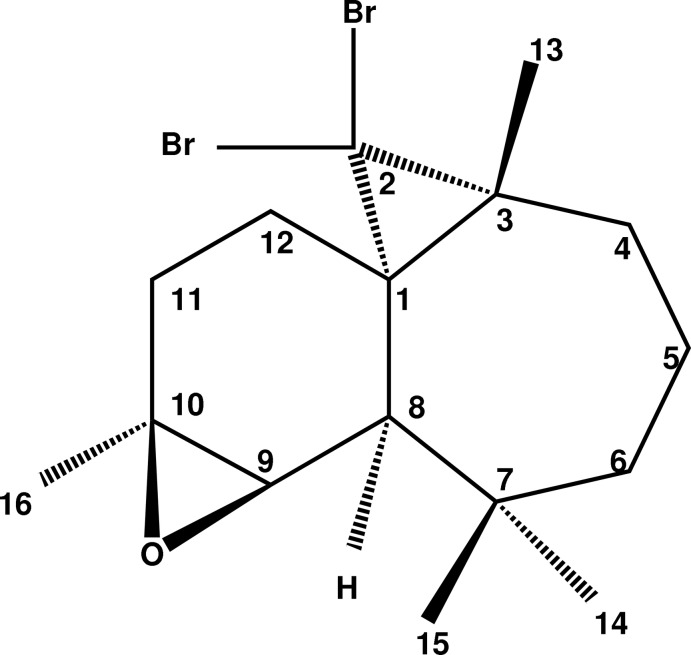


## Experimental

### 

#### Crystal data

C_16_H_24_Br_2_O
*M* *_r_* = 392.17
Orthorhombic, 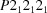

*a* = 7.9772 (4) Å
*b* = 12.8562 (7) Å
*c* = 16.1719 (8) Å
*V* = 1658.53 (15) Å^3^
*Z* = 4
Mo *K*α radiation
μ = 4.88 mm^−1^
*T* = 296 K
0.41 × 0.32 × 0.27 mm

#### Data collection

Bruker X8 APEX diffractometer
Absorption correction: multi-scan (*SADABS*; Sheldrick, 2008 ▶) *T*_min_ = 0.407, *T*_max_ = 0.747
15200 measured reflections
4637 independent reflections
3298 reflections with *I* > 2σ(*I*)
*R*_int_ = 0.036

#### Refinement

*R*[*F* ^2^ > 2σ(*F* ^2^)] = 0.033
*wR*(*F* ^2^) = 0.074
*S* = 1.02
4637 reflections
173 parameters
H-atom parameters constrained
Δρ_max_ = 0.41 e Å^−3^
Δρ_min_ = −0.46 e Å^−3^
Absolute structure: Flack & Bernardinelli (2000 ▶), 1998 Friedel pairs
Flack parameter: 0.014 (10)

### 

Data collection: *APEX2* (Bruker, 2009 ▶); cell refinement: *SAINT* (Bruker, 2009 ▶); data reduction: *SAINT*; program(s) used to solve structure: *SHELXS97* (Sheldrick, 2008 ▶); program(s) used to refine structure: *SHELXL97* (Sheldrick, 2008 ▶); molecular graphics: *ORTEP-3 for Windows* (Farrugia, 2012 ▶); software used to prepare material for publication: *PLATON* (Spek, 2009 ▶) and *publCIF* (Westrip, 2010 ▶).

## Supplementary Material

Crystal structure: contains datablock(s) I, global. DOI: 10.1107/S1600536813006077/fj2620sup1.cif

Structure factors: contains datablock(s) I. DOI: 10.1107/S1600536813006077/fj2620Isup2.hkl

Supplementary material file. DOI: 10.1107/S1600536813006077/fj2620Isup3.cml

Additional supplementary materials:  crystallographic information; 3D view; checkCIF report

## Figures and Tables

**Table 1:** Hydrogen-bond geometry (Å, °)

*D*—H⋯*A*	*D*—H	H⋯*A*	*D*⋯*A*	*D*—H⋯*A*
C9—H9⋯O1^i^	0.98	2.53	3.391 (3)	146

Symmetry code: (i) 
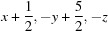
.

**Fig. 1.**
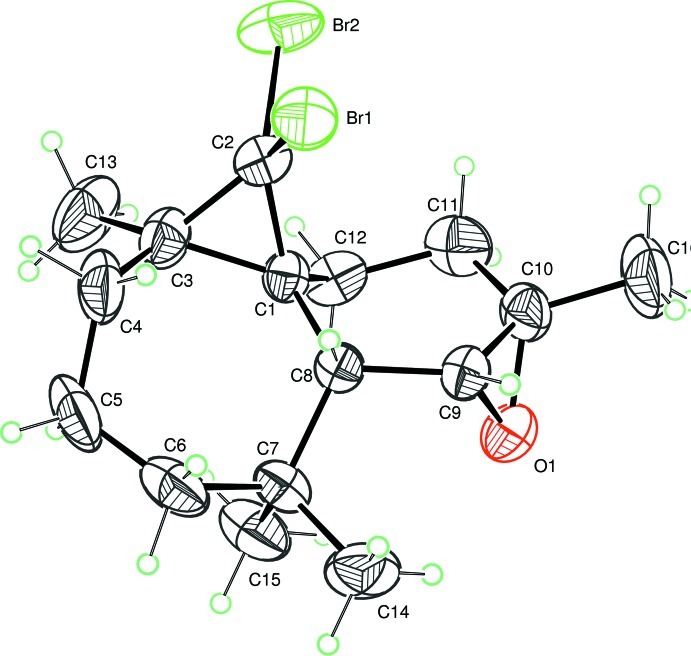
Molecular structure of the title compound with the atom-labelling scheme. Displacement ellipsoids are drawn at the 50% probability level. H atoms are represented as small spheres of arbitrary radii.

**Fig. 2.**
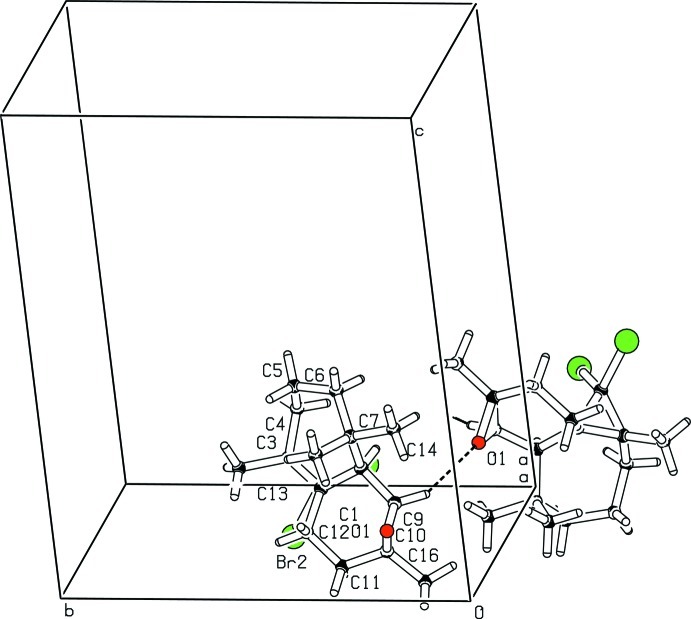
Molecule and its symmetry partner linked by C9–H9···O1 non classic hydrogen bond. Symmetry codes: (i) 1 + x, -1 + y, z.

## supplementary crystallographic information

### Comment

Our work lies within the framework of the valorization of the most abundant essential oils in Morocco, such as Cedrus atlantica. This oil is made up mainly (75%) of bicyclic sesquiterpenes hydrocarbons, among which is found the compound, β-himachalene (Joseph & Dev, 1968; Plattier & Teisseire, 1974). The reactivity of this sesquiterpene and its derivatives has been studied extensively by our team in order to prepare new products having biological proprieties (Lassaba *et al.*, 1998; Chekroun *et al.*, 2000; El Jamili *et al.*, 2002; Sbai *et al.*, 2002; Dakir *et al.*, 2004). Indeed, these compounds were tested, using the food poisoning technique, for their potential antifungal activity against phytopathogen Botrytis cinerea (Daoubi *et al.*, 2004). Thus the action of one equivalent of dibromocabene, generated *in situ* from bromoform in the presence of sodium hydroxide as base and n-benzyltriethylammonium chloride as catalyst, on β-himachalene produces only (1*S*,3*R*,8*R*)-2,2-dibromo-3,7,7,10- tetramethyltricyclo[6.4.0.0^1,3^]dodec-9-ene (El Jamili *et al.*, 2002). Treatment of the latter by one equivalent of *m*-chloroperbenzoic acid (*m*-CPBA) gives a mixture of two diastereoisomers: (1*S*,3*R*,8*R*,9S,10*R*)-2,2-dibromo-9α,10α-epoxy- 3,7,7,10- tetramethyltricyclo-[6.4.0.0^1,3^]dodecane (*X*) and its isomer (1*S*,3*R*,8*R*,9*R*,10*S*)-2,2-dibromo)- 9β,10β-epoxy-3,7,7,10-Tetramethyltricyclo-[6.4.0.0^1,3^]dodecane (*Y*) in an over-all yield of 65% and 15/85 ratio. By single-crystal X-ray diffraction analysis, we have determined the absolute configuration of *Y* and we deduced that from its isomer *X*.

The molecule contains a fused six- and seven-membered rings, which is fused to two three-membered rings as shown in Fig.1. The six-membered ring has a half chair conformation as indicated by the total puckering amplitude QT = 0.520 (3) Å and spherical polar angle θ = 53.6 (3)° with φ2 = -97.9 (4)°, whereas the seven-membered ring displays a chair conformation with QT = 0.7961 (3) Å, θ2 = 32.4 (2)°, φ2 = -51.9 (4)° and φ3 = -78.7 (2)° (Cremer & Pople, 1975). The dihedral angle between the six and seven-membered rings is 59.3 (2)°. The three-membered rings (C1C2C3) and (C9O1C10) are nearly perpendicular to the six-membered ring (C1C8C9C11C12C13) with a dihedral angle of 78.6 (2)° and 80.5 (2)°, respectively. Owing to the presence of Br atoms, the absolute configuration could be fully confirmed from anomalous dispersion effects, by refining the Flack parameter (Flack & Bernardinelli (2000)) as C1(S), C3(*R*), C8(*R*), C9(S), and C10(*R*).

In the crystal, each molecule is linked to its symmetry equivalent partner by C9–H9···O1 non classic hydrogen-bond as shown in Fig.2 and Table 2. The present structure is similar to that of C_16_H_24_OCl_2_ published, in a previous work, by Benharref *et al.* (2010).

### Experimental

For the synthesis of compounds (1*S*,3*R*,8*S*,9S,10*R*)-2,2-dibromo-9α,10α-epoxy- 3,7,7,10-tetramethyltricyclo [6.4.0.0^1,3^]dodecane (*X*) and its isomer (1*S*,3*R*,8*S*,9*R*,10*S*)-2,2-dibromo-9β,10β-epoxy-3,7,7,10-tetramethyltricyclo [6.4.0.0^1,3^]dodecane (Y), a stoichiometric quantity of *m*-chloroperbenzoic acid (*m*-CPBA) was added to a 250 ml flask containing a solution of (1*S*,3*R*,8*S*)-2,2-dibromo-3,7,7,10- tetramethyltricyclo[6,4,0,0^1,3^] dodec-9-ene (2 g, 5.3 mmol) in dichloromethane (100 ml). The reaction mixture was stirred at ambient temperature for 2 h, then treated with a 10% solution of sodium hydrogencarbonate. The aqueous phase was extracted with dichloromethane and the organic phases were dried and concentrated.The residue obtained was chromatographed on silica gel column impregnated with silver nitrate (10%) with a mixture of hexane - ethyl acetate (98–2) used as eluent. The two diastereoisomers: (1*S*,3*R*,8*R*,9S,10*R*)-2,2-dibromo-9α,10α-epoxy- 3,7,7,10-tetramethyltricyclo-[6.4.0.01,3]dodecane (*X*) and its isomer (1*S*,3*R*,8*R*,9*R*,10*S*)-2,2-dibromo)-9β,10β-epoxy-3,7,7,10-Tetramethyl tricyclo-[6.4.0.01,3]dodecane (*Y*) were obtained by this procedure in a 15/85 ratio and a combined yield of 65% (1.35 g; 3.4 mmol). The title compound (isomer *Y*) was recrystallized from hexane.

### Refinement

All H atoms were fixed geometrically and treated as riding with C—H = 0.96 Å (methyl),0.97 Å (methylene), 0.98 Å (methine) with *U*_iso_(H) = 1.2Ueq(methylene, methine) or *U*_iso_(H) = 1.5Ueq(methyl). The space group is not centro symmetric and the polar axis restraint is generated automatically by *SHELXL* program. The Friedel opposites reflections are not merged.

### Crystal data

**Table:**

C_16_H_24_Br_2_O	*F*(000) = 792
*M**_r_* = 392.17	*D*_x_ = 1.571 Mg m^−^^3^
Orthorhombic, *P*2_1_2_1_2_1_	Mo *K*α radiation, λ = 0.71073 Å
Hall symbol: p 2ac 2ab	Cell parameters from 4637 reflections
*a* = 7.9772 (4) Å	θ = 2.9–29.6°
*b* = 12.8562 (7) Å	µ = 4.88 mm^−^^1^
*c* = 16.1719 (8) Å	*T* = 296 K
*V* = 1658.53 (15) Å^3^	Block, colourless
*Z* = 4	0.41 × 0.32 × 0.27 mm

### Data collection

**Table:**

Bruker X8 APEX diffractometer	4637 independent reflections
Radiation source: fine-focus sealed tube	3298 reflections with *I* > 2σ(*I*)
Graphite monochromator	*R*_int_ = 0.036
φ and ω scans	θ_max_ = 29.6°, θ_min_ = 2.9°
Absorption correction: multi-scan (*SADABS*; Sheldrick, 2008)	*h* = −11→11
*T*_min_ = 0.407, *T*_max_ = 0.747	*k* = −17→15
15200 measured reflections	*l* = −22→14

### Refinement

**Table:**

Refinement on *F*^2^	Hydrogen site location: inferred from neighbouring sites
Least-squares matrix: full	H-atom parameters constrained
*R*[*F*^2^ > 2σ(*F*^2^)] = 0.033	*w* = 1/[σ^2^(*F*_o_^2^) + (0.0307*P*)^2^] where *P* = (*F*_o_^2^ + 2*F*_c_^2^)/3
*wR*(*F*^2^) = 0.074	(Δ/σ)_max_ = 0.001
*S* = 1.02	Δρ_max_ = 0.41 e Å^−^^3^
4637 reflections	Δρ_min_ = −0.46 e Å^−^^3^
173 parameters	Extinction correction: *SHELXL97* (Sheldrick, 2008), Fc^*^=kFc[1+0.001xFc^2^λ^3^/sin(2θ)]^-1/4^
0 restraints	Extinction coefficient: 0.0016 (5)
Primary atom site location: structure-invariant direct methods	Absolute structure: Flack & Bernardinelli (2000), 1998 Friedel pairs
Secondary atom site location: difference Fourier map	Flack parameter: 0.014 (10)

### Special details

**Table:**

Geometry. All e.s.d.'s (except the e.s.d. in the dihedral angle between two l.s. planes) are estimated using the full covariance matrix. The cell e.s.d.'s are taken into account individually in the estimation of e.s.d.'s in distances, angles and torsion angles; correlations between e.s.d.'s in cell parameters are only used when they are defined by crystal symmetry. An approximate (isotropic) treatment of cell e.s.d.'s is used for estimating e.s.d.'s involving l.s. planes.
Refinement. Refinement of *F*^2^ against all reflections. The weighted *R*-factor *wR* and goodness of fit *S* are based on *F*^2^, conventional *R*-factors *R* are based on *F*, with *F* set to zero for negative *F*^2^. The threshold expression of *F*^2^ > σ(*F*^2^) is used only for calculating *R*-factors(gt) *etc*. and is not relevant to the choice of reflections for refinement. *R*-factors based on *F*^2^ are statistically about twice as large as those based on *F*, and *R*- factors based on all data will be even larger.

### Fractional atomic coordinates and isotropic or equivalent isotropic displacement parameters (Å^2^)

**Table:**

	*x*	*y*	*z*	*U*_iso_*/*U*_eq_	
C1	0.4959 (3)	0.9764 (2)	0.00560 (15)	0.0325 (5)	
C2	0.6592 (3)	0.9395 (2)	0.04293 (16)	0.0404 (6)	
C3	0.5934 (3)	0.8863 (2)	−0.03369 (19)	0.0448 (7)	
C4	0.6887 (4)	0.9048 (3)	−0.1148 (2)	0.0587 (9)	
H4A	0.7562	0.8439	−0.1270	0.070*	
H4B	0.7641	0.9633	−0.1076	0.070*	
C5	0.5722 (4)	0.9266 (3)	−0.1883 (2)	0.0676 (11)	
H5A	0.6300	0.9089	−0.2392	0.081*	
H5B	0.4741	0.8824	−0.1841	0.081*	
C6	0.5167 (4)	1.0390 (3)	−0.19240 (18)	0.0596 (9)	
H6A	0.4619	1.0491	−0.2454	0.072*	
H6B	0.6169	1.0816	−0.1927	0.072*	
C7	0.3993 (3)	1.0825 (2)	−0.12543 (16)	0.0433 (7)	
C8	0.4885 (3)	1.0819 (2)	−0.03933 (15)	0.0316 (5)	
H8	0.6055	1.1004	−0.0506	0.038*	
C9	0.4239 (3)	1.1658 (2)	0.01801 (16)	0.0374 (6)	
H9	0.4807	1.2330	0.0124	0.045*	
O1	0.2442 (2)	1.17299 (17)	0.03278 (13)	0.0503 (5)	
C10	0.3546 (3)	1.1457 (2)	0.10036 (16)	0.0454 (7)	
C11	0.3355 (3)	1.0354 (3)	0.12860 (17)	0.0540 (8)	
H11A	0.2298	1.0284	0.1577	0.065*	
H11B	0.4247	1.0193	0.1673	0.065*	
C12	0.3405 (3)	0.9577 (2)	0.05873 (17)	0.0434 (6)	
H12A	0.2405	0.9648	0.0251	0.052*	
H12B	0.3430	0.8877	0.0810	0.052*	
C13	0.5257 (5)	0.7765 (3)	−0.0288 (3)	0.0738 (11)	
H13A	0.4902	0.7545	−0.0828	0.111*	
H13B	0.4320	0.7747	0.0084	0.111*	
H13C	0.6119	0.7307	−0.0090	0.111*	
C14	0.3664 (5)	1.1961 (3)	−0.1496 (2)	0.0664 (9)	
H14A	0.2932	1.2275	−0.1097	0.100*	
H14B	0.3149	1.1984	−0.2032	0.100*	
H14C	0.4707	1.2333	−0.1511	0.100*	
C15	0.2311 (3)	1.0239 (3)	−0.12733 (19)	0.0626 (9)	
H15A	0.2492	0.9523	−0.1129	0.094*	
H15B	0.1839	1.0279	−0.1818	0.094*	
H15C	0.1552	1.0548	−0.0884	0.094*	
C16	0.3686 (5)	1.2270 (3)	0.1671 (2)	0.0763 (11)	
H16A	0.4449	1.2034	0.2090	0.115*	
H16B	0.2602	1.2387	0.1913	0.115*	
H16C	0.4094	1.2907	0.1436	0.115*	
Br1	0.85914 (3)	1.02421 (3)	0.041525 (19)	0.05303 (11)	
Br2	0.66225 (4)	0.86201 (3)	0.14535 (2)	0.07179 (14)	

### Atomic displacement parameters (Å^2^)

**Table:**

	*U*^11^	*U*^22^	*U*^33^	*U*^12^	*U*^13^	*U*^23^
C1	0.0318 (11)	0.0302 (14)	0.0355 (13)	−0.0019 (12)	0.0017 (10)	−0.0015 (12)
C2	0.0370 (12)	0.0395 (15)	0.0446 (14)	0.0013 (12)	−0.0010 (13)	0.0059 (12)
C3	0.0425 (14)	0.0336 (17)	0.0584 (17)	0.0003 (11)	−0.0005 (13)	−0.0066 (14)
C4	0.0548 (17)	0.059 (2)	0.0627 (19)	0.0102 (15)	0.0065 (15)	−0.0287 (17)
C5	0.074 (2)	0.086 (3)	0.0422 (17)	−0.001 (2)	0.0017 (16)	−0.0345 (18)
C6	0.0609 (18)	0.088 (3)	0.0302 (14)	−0.002 (2)	−0.0040 (12)	−0.0032 (16)
C7	0.0497 (15)	0.0508 (19)	0.0295 (13)	−0.0009 (13)	−0.0044 (11)	−0.0007 (12)
C8	0.0321 (11)	0.0323 (14)	0.0303 (12)	−0.0011 (10)	0.0037 (11)	0.0021 (11)
C9	0.0376 (12)	0.0331 (17)	0.0415 (15)	0.0005 (11)	0.0017 (11)	−0.0021 (12)
O1	0.0408 (9)	0.0570 (14)	0.0532 (12)	0.0160 (9)	0.0026 (9)	−0.0053 (11)
C10	0.0388 (13)	0.0579 (19)	0.0397 (14)	0.0083 (15)	0.0041 (13)	−0.0110 (13)
C11	0.0461 (15)	0.075 (2)	0.0411 (15)	0.0023 (17)	0.0126 (13)	0.0073 (15)
C12	0.0327 (12)	0.0433 (17)	0.0543 (16)	−0.0012 (13)	0.0049 (12)	0.0120 (13)
C13	0.066 (2)	0.033 (2)	0.123 (4)	−0.0004 (16)	−0.013 (2)	−0.007 (2)
C14	0.079 (2)	0.071 (2)	0.0487 (17)	0.011 (2)	−0.0039 (19)	0.0207 (17)
C15	0.0542 (16)	0.090 (3)	0.0436 (16)	−0.0074 (19)	−0.0102 (13)	−0.0064 (19)
C16	0.076 (2)	0.089 (3)	0.064 (2)	0.012 (2)	0.0117 (19)	−0.039 (2)
Br1	0.03457 (13)	0.0605 (2)	0.06396 (19)	−0.00414 (15)	−0.00528 (13)	−0.00561 (16)
Br2	0.0688 (2)	0.0793 (3)	0.0673 (2)	0.0137 (2)	−0.00527 (18)	0.03512 (19)

### Geometric parameters (Å, º)

**Table:**

C1—C2	1.512 (3)	C9—O1	1.457 (3)
C1—C12	1.527 (3)	C9—C10	1.465 (4)
C1—C3	1.533 (4)	C9—H9	0.9800
C1—C8	1.540 (4)	O1—C10	1.447 (3)
C2—C3	1.510 (4)	C10—C11	1.498 (4)
C2—Br1	1.931 (3)	C10—C16	1.507 (4)
C2—Br2	1.933 (3)	C11—C12	1.508 (4)
C3—C13	1.513 (5)	C11—H11A	0.9700
C3—C4	1.534 (4)	C11—H11B	0.9700
C4—C5	1.535 (5)	C12—H12A	0.9700
C4—H4A	0.9700	C12—H12B	0.9700
C4—H4B	0.9700	C13—H13A	0.9600
C5—C6	1.512 (6)	C13—H13B	0.9600
C5—H5A	0.9700	C13—H13C	0.9600
C5—H5B	0.9700	C14—H14A	0.9600
C6—C7	1.537 (4)	C14—H14B	0.9600
C6—H6A	0.9700	C14—H14C	0.9600
C6—H6B	0.9700	C15—H15A	0.9600
C7—C14	1.534 (5)	C15—H15B	0.9600
C7—C15	1.540 (4)	C15—H15C	0.9600
C7—C8	1.564 (3)	C16—H16A	0.9600
C8—C9	1.513 (4)	C16—H16B	0.9600
C8—H8	0.9800	C16—H16C	0.9600
			
C2—C1—C12	115.2 (2)	O1—C9—C8	118.8 (2)
C2—C1—C3	59.43 (18)	C10—C9—C8	124.1 (2)
C12—C1—C3	121.8 (2)	O1—C9—H9	114.5
C2—C1—C8	119.8 (2)	C10—C9—H9	114.5
C12—C1—C8	111.9 (2)	C8—C9—H9	114.5
C3—C1—C8	119.3 (2)	C10—O1—C9	60.61 (16)
C3—C2—C1	60.96 (18)	O1—C10—C9	60.03 (16)
C3—C2—Br1	122.22 (19)	O1—C10—C11	113.5 (2)
C1—C2—Br1	122.02 (19)	C9—C10—C11	118.8 (2)
C3—C2—Br2	118.3 (2)	O1—C10—C16	114.7 (3)
C1—C2—Br2	120.94 (17)	C9—C10—C16	120.1 (3)
Br1—C2—Br2	106.89 (12)	C11—C10—C16	116.5 (3)
C2—C3—C13	120.3 (3)	C10—C11—C12	113.3 (2)
C2—C3—C1	59.61 (18)	C10—C11—H11A	108.9
C13—C3—C1	120.1 (3)	C12—C11—H11A	108.9
C2—C3—C4	117.3 (2)	C10—C11—H11B	108.9
C13—C3—C4	111.4 (3)	C12—C11—H11B	108.9
C1—C3—C4	119.3 (3)	H11A—C11—H11B	107.7
C3—C4—C5	113.0 (3)	C11—C12—C1	109.8 (2)
C3—C4—H4A	109.0	C11—C12—H12A	109.7
C5—C4—H4A	109.0	C1—C12—H12A	109.7
C3—C4—H4B	109.0	C11—C12—H12B	109.7
C5—C4—H4B	109.0	C1—C12—H12B	109.7
H4A—C4—H4B	107.8	H12A—C12—H12B	108.2
C6—C5—C4	112.7 (3)	C3—C13—H13A	109.5
C6—C5—H5A	109.0	C3—C13—H13B	109.5
C4—C5—H5A	109.0	H13A—C13—H13B	109.5
C6—C5—H5B	109.0	C3—C13—H13C	109.5
C4—C5—H5B	109.0	H13A—C13—H13C	109.5
H5A—C5—H5B	107.8	H13B—C13—H13C	109.5
C5—C6—C7	119.7 (3)	C7—C14—H14A	109.5
C5—C6—H6A	107.4	C7—C14—H14B	109.5
C7—C6—H6A	107.4	H14A—C14—H14B	109.5
C5—C6—H6B	107.4	C7—C14—H14C	109.5
C7—C6—H6B	107.4	H14A—C14—H14C	109.5
H6A—C6—H6B	106.9	H14B—C14—H14C	109.5
C14—C7—C6	105.7 (3)	C7—C15—H15A	109.5
C14—C7—C15	108.2 (3)	C7—C15—H15B	109.5
C6—C7—C15	109.8 (3)	H15A—C15—H15B	109.5
C14—C7—C8	108.1 (2)	C7—C15—H15C	109.5
C6—C7—C8	110.4 (2)	H15A—C15—H15C	109.5
C15—C7—C8	114.3 (2)	H15B—C15—H15C	109.5
C9—C8—C1	110.6 (2)	C10—C16—H16A	109.5
C9—C8—C7	112.7 (2)	C10—C16—H16B	109.5
C1—C8—C7	116.3 (2)	H16A—C16—H16B	109.5
C9—C8—H8	105.4	C10—C16—H16C	109.5
C1—C8—H8	105.4	H16A—C16—H16C	109.5
C7—C8—H8	105.4	H16B—C16—H16C	109.5
O1—C9—C10	59.36 (18)		

### Hydrogen-bond geometry (Å, º)

**Table:**

*D*—H···*A*	*D*—H	H···*A*	*D*···*A*	*D*—H···*A*
C9—H9···O1^i^	0.98	2.53	3.391 (3)	146

Symmetry code: (i) *x*+1/2, −*y*+5/2, −*z*.
